# Virus Control Goes Epigenetic

**DOI:** 10.1371/journal.ppat.1004370

**Published:** 2014-09-11

**Authors:** Jing-hsiung James Ou

**Affiliations:** Department of Molecular Microbiology and Immunology, University of Southern California, Keck School of Medicine, Los Angeles, California, United States of America; The Fox Chase Cancer Center, United States of America

Cells have developed a number of mechanisms to detect and suppress microbial infections. The use of pattern recognition receptors (PRRs) is one such mechanism. PRRs may be expressed on the cell surface, in the cytosol, or in the endosomes, to detect pathogen-associated molecular patterns (PAMPs) or the danger signals released by damaged cells (i.e., damage-associated molecular patterns; DAMPs). PRR activation triggers a signaling cascade, resulting in the production of antimicrobial cytokines, including type I interferons. These cytokines will in turn induce the expression of genes, such as interferon-stimulated genes (ISGs), that exhibit antimicrobial activities. In addition to PRRs, autophagy is another intracellular defense mechanism. Autophagy is used by the cells to remove intracellular pathogens in a process known as xenophagy [1]. Many viruses have developed different mechanisms to suppress these innate immune responses for their survival. A notable example is the cleavage of the mitochondrial antiviral signaling protein (MAVS) by the hepatitis C virus (HCV) NS3 protease [2]. MAVS is an important downstream adaptor molecule of RIG-I, which is a cytosolic PRR that can be activated by the HCV genomic RNA. Another example is the influenza virus NS1 protein, which binds to the E3 ubiquitin ligase TRIM25 to prevent it from activating RIG-I [3]. Similarly, the autophagy pathway may also be suppressed by viruses or even exploited by viruses to benefit their own replication. The former is typified by the herpes simplex virus-1 (HSV-1) ICP34.5 protein, which binds to Beclin-1 to prevent it from binding to and activating the class III phosphatidylinositol-3-kinase, an enzyme important for the initiation of autophagy. An example of the latter is HCV, which uses autophagy to enhance its replication [4].

In the report by Ducroux et al. [5], the authors presented yet another type of cellular control of viral infections. By using a tandem-affinity purification approach, the authors identified Spindlin1 as a binding partner of the hepatitis B virus (HBV) X protein (HBx). They subsequently discovered that Spindlin1 could suppress the replication of HBV, as its overexpression reduced the levels of HBV RNA and DNA replicative intermediates. On the contrary, depletion of Spindlin1 by RNA interference enhanced HBV replication. They then demonstrated that the suppressive effect of Spindlin1 on HBV was at the transcriptional level, as the overexpression of Spindlin1 reduced the transcriptional activity of HBV DNA by 80% in a nuclear run-on experiment. This effect of Spindlin1 on HBV was specific, as it was not observed with the cellular gene cyclin A2.

Spindlin1 contains three Tudor-like domains and can bind to trimethylated lysine 4 (H3K4me3) and asymmetric dimethylated arginine 8 (H3R8me2a) of histone H3 [6]. In the chromatin immunoprecipitation (ChIP) assay, Ducroux et al. demonstrated that Spindlin1 could bind to the HBV covalently closed circular DNA (cccDNA) genome. Interestingly, this binding was enhanced if the expression of HBx from the viral genome was abolished, suggesting an inhibitory role of HBx in the binding of Spindlin1 to the HBV genome. It does not appear likely that Spindlin1 binds to HBV cccDNA via H3K4me3, though, as the depletion of Spindlin1 actually led to a significant increase of the level of H3K4me3 in the HBV cccDNA. Their results suggested that Spindlin1, by binding to the HBV cccDNA, inhibits this post-translational modification of histone H3, which is associated with actively transcribed genes (Figure 1) [7].

**Figure 1:**
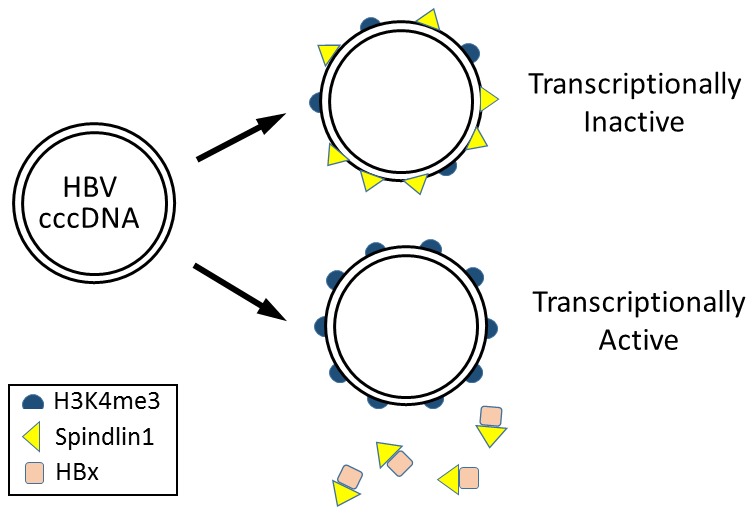
Model for epigenetic regulation of HBV gene expression by Spindlin1. HBV cccDNA in the nucleus is associated with nucleosomes, which may be modified by trimethylation at lysine 4 of histone H3 (H3K4me3). Spindlin1 binds the HBV cccDNA to reduce the H3K4me3 level, resulting in the suppression of HBV gene expression. In the presence of HBx, Spindlin1 is sequestered from the cccDNA and the level of H3K4me3 increases, leading to the activation of HBV gene expression.

A similar suppressive effect of Spindlin1 on the transcription of HSV-1 RNA was also observed by the authors. They found that the depletion of Spindlin1 also increased the ICP27 RNA level of HSV-1. In contrast, Spindlin1 had no effect on the replication of HCV, an RNA virus. Their results raise the possibility that Spindlin1, either by itself or by interacting with an unknown partner or partners, may represent a new class of PRRs that detect and suppress gene expression of DNA viruses by reducing the H3K4me3 level on the viral genome. If Spindlin1 is indeed a new PRR, then it will be interesting to determine how Spindlin1 differentiates episomal viral DNA from self DNA and what PAMPs it recognizes on the viral DNA. In their studies on HSV-1, Ducroux et al. did not demonstrate whether Spindlin1 could indeed reduce the H3K4me3 level associated with the HSV-1 genome, which must be demonstrated if the ability of Spindlin1 to exert epigenetic control on gene expression of DNA viruses is to be generalized. It will also be interesting in the future to determine whether Spindlin1 exhibits the same suppressive effect on other DNA viruses such as papillomaviruses and polyomaviruses and whether these DNA viruses also express gene products that can antagonize the suppressive effect of Spindlin1.

## References

[1] Dong X, Levine B (2013) Autophagy and viruses: adversaries or allies? J Innate Immun 5: 480–493. 23391695 10.1159/000346388 PMC3790331

[2] Horner SM, Gale M Jr (2013) Regulation of hepatic innate immunity by hepatitis C virus. Nat Med 19: 879–888. 23836238 10.1038/nm.3253 PMC4251871

[3] Gack MU, Albrecht RA, Urano T, Inn KS, Huang IC, et al (2009) Influenza A virus NS1 targets the ubiquitin ligase TRIM25 to evade recognition by the host viral RNA sensor RIG-I. Cell Host Microbe 5: 439–449. 19454348 10.1016/j.chom.2009.04.006 PMC2737813

[4] Sir D, Ou JH (2010) Autophagy in viral replication and pathogenesis. Mol Cells 29: 1–7. 20077024 10.1007/s10059-010-0014-2 PMC3115743

[5] Ducroux A, Benhenda S, Riviere L, Semmes OJ, Benkirane M, et al (2014) The Tudor domain protein Spindlin1 is involved in intrinsic antiviral defense against incoming hepatitis B virus and herpes simplex virus type 1. PLoS Pathog 10: e1004343. 25211330 10.1371/journal.ppat.1004343 PMC4161474

[6] Su X, Zhu G, Ding X, Lee SY, Dou Y, et al (2014) Molecular basis underlying histone H3 lysine-arginine methylation pattern readout by Spin/Ssty repeats of Spindlin1. Genes Dev 28: 622–636. 24589551 10.1101/gad.233239.113 PMC3967050

[7] Santos-Rosa H, Schneider R, Bannister AJ, Sherriff J, Bernstein BE, et al (2002) Active genes are tri-methylated at K4 of histone H3. Nature 419: 407–411. 12353038 10.1038/nature01080

